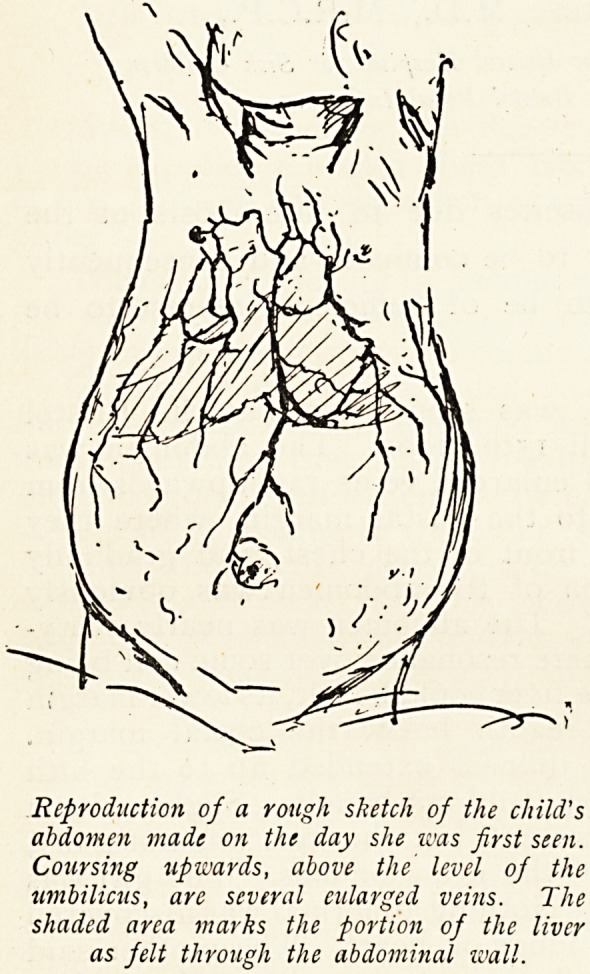# A Case of Ascites Due to Thrombosis of the Hepatic Veins

**Published:** 1902-09

**Authors:** Theodore Fisher

**Affiliations:** Physician to Out-Patients to the Bristol Hospital for Sick Children; Pathologist to the Bristol Royal Infirmary


### A CASE OF ASCITES DUE TO THROMBOSIS OF THE HEPATIC VEINS.

BY

Theodore Fisher, M.D., M.R.C.P.,

*Physician to Out-Patients to the Bristol Hospital for Sick Children; Pathologist to the Bristol Royal Infirmary.*

---

Recorded occurrences of ascites due to thrombosis of the hepatic veins do not appear to be common, and consequently the following case seems to be of sufficient interest to be worthy of publication:—

A girl, aged three years, was seen by me at the Bristol Children's Hospital on April 15th, 1901. The abdomen was much distended, and several enlarged veins ran upwards from the level of the umbilicus to the costal margin, where they formed a network over the front of the chest, and gradually disappeared. The distension of the abdomen was obviously due to the presence of fluid. The abdomen was nearly everywhere dull; only here and there resonance over some coil being obtained. By "bobbing" the liver could be felt, its lower margin extending to four fingers' breadth below the costal margin. In the right axilla the liver dulness extended up to the fifth intercostal space. The spleen could not be felt. Slight œdema of the thighs and legs was present, but the face was not puffy, and there was no swelling of the arms or legs. The girl was pale, but did not look very ill. Nothing abnormal was discovered in the physical signs of the lungs or heart. The mother said the child had been well up till about five months before, when she was attacked with whooping cough, which lasted three or four months. The swelling of the abdomen had been noticed about one month. There had been no diarrhœa: the bowels had generally been somewhat constipated. The child passed little urine, but nothing had been noticed abnormal in its colour. There had been eight other children in the family: two had died—one from bronchitis after measles, the other as a baby. The father had died six months before of Bright's disease. Cirrhosis of the liver was thought of as a possible cause for the enlargement of the liver and ascites; but there was nothing about the child to suggest syphilis, and the mother denied that she had been given alcoholic drinks. The mother would not consent to leave the child at the hospital, but three weeks later she took her to the Bristol Royal Infirmary, where she was admitted under the care of Dr. Shingleton Smith. On May 7th, the day after admission, the abdomen was tapped, and 65 ounces of fluid drawn off. The child, however, did not improve; the abdomen refilled; she suffered from two severe attacks of epistaxis. Diarrhœa set in, and during the last fortnight of life there was moderate pyrexia. She died June 6th.

At the autopsy the body was much emaciated. The skin was of yellowish tint. The peritoneal cavity contained about 60 ounces of slightly reddish-brown coloured fluid. The liver was enlarged; it weighed 23 ounces. The anterior third of the upper surface was slightly granular, but elsewhere smooth. On section it was somewhat bile-stained, and extremely "nutmegged." On closer inspection the larger divisions of the hepatic veins were seen to be thrombosed. The contained clot was pale, and the walls of the veins thickened. Both hepatic veins, where they should have opened into the vena cava, were found to be completely blocked. A small fibroid nodule marked the site of the entrance of one vein, and a small depression the size of a pin's head that of the other. A vein, the size of an ordinary pen-holder, ran from the left branch of the portal vein downwards in the falciform ligament to the umbilicus. The stomach contained several ounces of dark, semi-digested blood, but no local cause, not even a small hemorrhagic erosion, could be found to account for it. The œsophageal veins were little if at all dilated. The spleen was not enlarged; it weighed one ounce. There was nothing noteworthy in any of the other organs. Cultures taken from the liver and thrombi shewed the presence of streptococci, but they probably were merely the evidence of a terminal infection and explained the pyrexia which had been present during the last two weeks of life. Sections of the liver shewed the hepatic veins to be completely obliterated where they joined the inferior vena cava. Everywhere the walls of the main divisions of the veins were much thickened, and the clot they contained was in

*Reproduction of a rough sketch of the child's abdomen made on the day she was first seen. Coursing upwards, above the level of the umbilicus, are several enlarged veins. The shaded area marks the portion of the liver as felt through the abdominal wall.*

various stages of organisation. In some of them the liver-cells were largely replaced by dilated capillaries full of blood-cells, but in others pigmentation of the liver-cells and the presence of fat were the chief features. Here and there the interstitial tissue surrounding the medium-sized divisions of the portal vein was increased, but the cirrhosis was slight in degree.

As has been already mentioned, thrombosis of the hepatic veins does not appear to be common, yet possibly it may be sometimes overlooked. I have met with two other comparatively unimportant examples. In one the thrombosis was associated with septic thrombosis of the portal vein, in the other the thrombosis of the hepatic veins occurred as a complication of broncho-pneumonia in a child. In the second case there was no ascites, but the liver was extremely congested and in places hepatic lobules had been completely destroyed by the pressure of the dilated venous capillaries.

Cases of thrombosis of the hepatic veins similar to the example reported above have been recorded by Gee,[1] Churton,[2] Rolleston,[3] Lazarus-Barlow,[4] and Kelynack.[5] Gee refers also to a case recorded by Von Recklinghausen, and Kelynack to one by Frerichs. A case probably of similar nature is also recorded by Willcocks.[6] In this case thrombosis of the vena cava was present from "the lower border of the liver to the diaphragm." The hepatic veins are not mentioned, but it is possible that the thrombosis may have originated in them. In the case of Frerichs, in addition to the thrombosis of the hepatic veins, the orifices of these veins were completely blocked. A nodule marked the site of the opening of one vein, and the other terminated "in a blind extremity." In Gee's case the orifices of the veins were represented by "shallow dimples." In the case of Churton the orifices were "very narrow," while in that of Kelynack the opening of only one vein could be found. In my own case, like that of Frerichs, a nodule marked the site of the opening of one vein, a shallow depression that of the other. Cirrhosis of the liver has been present in the

1 *St. Barth. Hosp. Rep.*, 1871, vii. 144.
2 *Tr. Path. Soc. Lond.*, 1899, L. 145. 3 *Ibid.*, 148.
4 *Ibid.*, 146. 5 *Med. Press Circ.*, 1897, [cxiv.] 633.
6 *Tr. Path. Soc. Lond.*, 1896, xlvii. 67.

majority of cases, but in some does not appear to have been very well-marked. It was absent in Rolleston's case. The size of the spleen is referred to in only a few cases. It was of normal size in the case of Gee and in my own case. In the case of Willcox also, where thrombosis of the vena cava behind the liver must have blocked the orifices of the hepatic veins, the spleen was "not enlarged." In Kelynack's case, however, the spleen weighed 13 ounces.

Clinically, a striking feature in some of the cases has been the prominence of superficial abdominal veins. The abdomen was covered with "a plexus of distended veins" in the case of Lazarus-Barlow, and similar enlargement was present in the cases of Churton and Willcox. Distension of these veins was also present in my own case. In only one case, however, does there appear to be a reference to a vein running down in the umbilical ligament from the left branch of the portal vein, such as was present in the above case. The case in which a similar vein is mentioned is that of Gee. Bleeding into the alimentary tract, indicated by hematemesis and melœna or the discovery of blood in the stomach after death, was present in the cases of Rolleston, Lazarus-Barlow, and in my own. Ascites was present in all. It appears to have commenced, in cases where the time of onset is mentioned, from six weeks to four months before death.

Some writers appear to have thought that the thrombosis of these veins is probably due to syphilis. If we consider the thrombosis to have been secondary to cicatricial contraction of the orifices of the hepatic veins, we may think it possible also that syphilitic disease of the terminations of these veins was the cause of the contraction. The contraction, however, may have been secondary to the thrombosis. Thickening of the walls of veins, met with in association with thrombosis, is probably very often a sequence, not the cause, of the *ante-mortem* clotting. The frequency with which micro-organisms are found in thrombi suggests that most cases of thrombosis are probably due to an infection of the blood. The hepatic veins, however, are probably veins in which one would not expect infective thrombosis to arise. Possibly the clotting may be an indirect, rather than the direct, result of infection. Toxins produced in the liver, as the result of successful efforts in destroying some forms of micro-organisms in the portal circulation, may be the cause of the thrombosis.

In conclusion, I should wish to express my thanks to Dr. Shingleton Smith, under whose care the child was in the Bristol Royal Infirmary, for allowing me to publish the case.

## Figures and Tables

**Figure f1:**